# Interstitial Leydig Cell Tumorigenesis—Leptin and Adiponectin Signaling in Relation to Aromatase Expression in the Human Testis

**DOI:** 10.3390/ijms21103649

**Published:** 2020-05-21

**Authors:** Michal Duliban, Ewelina Gorowska-Wojtowicz, Waclaw Tworzydlo, Agnieszka Rak, Malgorzata Brzoskwinia, Izabella Krakowska, Jan K. Wolski, Malgorzata Kotula-Balak, Bartosz J. Płachno, Barbara Bilinska

**Affiliations:** 1 Department of Endocrinology, Institute of Zoology and Biomedical Research, Jagiellonian University in Krakow, Gronostajowa 9, 30-387 Krakow, Poland; michal.duliban@doctoral.uj.edu.pl (M.D.); malgorzata.brzoskwinia@doctoral.uj.edu.pl (M.B.); barbara.bilinska@uj.edu.pl (B.B.); 2 Developmental Biology and Invertebrate Morphology, Institute of Zoology and Biomedical Research, Jagiellonian University in Krakow, Gronostajowa 9, 30-387 Krakow, Poland; w.tworzydlo@uj.edu.pl; 3 Department of Physiology and Toxicology of Reproduction, Institute of Zoology and Biomedical Research, Jagiellonian University in Krakow, Gronostajowa 9, 30-387 Krakow, Poland; agnieszka.rak@uj.edu.pl; 4 University Centre of Veterinary Medicine, University of Agriculture in Krakow, Mickiewicza 24/28, 30-059 Krakow, Poland; Izabella.krakowska@urk.edu.pl; 5 nOvum Fertility Clinic, Bociania 13, 02-807 Warszawa, Poland; jkwolski@op.pl; 6 Department of Plant Cytology and Embryology, Institute of Botany, Jagiellonian University in Krakow, Gronostajowa 9, 30-387 Krakow, Poland; bartosz.plachno@uj.edu.pl

**Keywords:** adiponectin, aromatase, leptin, Leydig cell tumor, ultrastructure

## Abstract

Although epidemiological studies from the last years report an increase in the incidences of Leydig cell tumors (previously thought to be a rare disease), the biochemical characteristics of that tumor important for understanding its etiology, diagnosis, and therapy still remains not completely characterized. Our prior studies reported G-protein coupled estrogen receptor signaling and estrogen level disturbances in Leydig cell tumors. In addition, we found that expressions of multi-level-acting lipid balance- and steroidogenesis–controlling proteins including peroxisome proliferator-activated receptor are altered in this tumor. In order to get deeper into the other molecular mechanisms that regulate lipid homeostasis in the Leydig cell tumor, here we investigate the presence and expression of newly-described hormones responsible for lipid homeostasis balancing (leptin and adiponectin), together with expression of estrogen synthase (aromatase). Samples of Leydig cell tumors (*n* = 20) were obtained from patients (31–45 years old) and used for light and transmission electron microscopic, western blotting, and immunohistochemical analyses. In addition, body mass index (BMI) was calculated. In tumor mass, abundant lipid accumulation in Leydig cells and various alterations of Leydig cell shape, as well as the presence of adipocyte-like cells, were observed. Marked lipid content and various lipid droplet size, especially in obese patients, may indicate alterations in lipid homeostasis, lipid processing, and steroidogenic organelle function in response to interstitial tissue pathological changes. We revealed significantly increased expression of leptin, adiponectin and their receptors, as well as aromatase in Leydig cell tumors in comparison to control. The majority of patients (*n* = 13) were overweight as indicated by their BMI. Moreover, a significant increase in expression of phospholipase C (PLC), and kinases Raf, ERK which are part of adipokine transductional pathways, was demonstrated. These data expand our previous findings suggesting that in human Leydig cell tumors, estrogen level and signaling, together with lipid status, are related to each other. Increased BMI may contribute to certain biochemical characteristics and function of the Leydig cell in infertile patients with a tumor. In addition, altered adipokine-estrogen microenvironment can have an effect on proliferation, growth, and metastasis of tumor cells. We report here various targets (receptors, enzymes, hormones) controlling lipid balance and estrogen action in Leydig cell tumors indicating their possible usefulness for diagnostics and therapy.

## 1. Introduction

The discoveries of leptin and adiponectin were the first indications that adipose tissue is an endocrine organ [1]. Today it is known that these major adipokines maintain homeostasis of glucose, lipid, and energy, each contributing to the communication between the adipocyte and other tissues. The signals, in turn, interact with each other to regulate cellular processes. Leptin was the first recognized for its prominent action on the hypothalamus to control food intake, energy expenditure, and body weight [2]. In obese individuals, leptin does not adequately regulate energy expenditure [3]. These patients are referred to as “leptin resistant” due to low basal metabolic rates, despite high circulating leptin [4]. Adiponectin exerts potent effects on the central endocrine axis (brain structures) also regulating energy amount and consumption. Moreover, adiponectin is responsible for encouraging the “healthy” expansion of adipose tissue, thereby preventing ectopic lipid accumulation [5]. Intravenous administration of adiponectin to leptin-deficient mice decreases their thermogenesis and weight [6].

The release of both leptin and adiponectin can be acutely enhanced through a variety of factors [7]. Circulating levels of leptin are proportional to fat mass and number of leptin receptors abundantly expressed in many tissues. It has been demonstrated that the biological activity of leptin strongly depends on its proper interactions with obesity receptors (Ob-R) [8]. Interestingly, adiponectin was originally identified in stomach extracts of cattle as the endogenous ligand of the orphan G protein-coupled receptor APJ [9].

Lipid homeostasis is crucial for function of the endocrine reproductive organs. However, in contrary to the female, not much is known still about the distinct role of adopokines in the male. Recently, adiponectin supervision of lipid accumulation and metabolism in lipid-rich tissues was linked to human reproductive function [7]. It was shown that leptin through its hypothalamic level directly effects on androgen synthesis [10]. In obese patients, increased leptin level was associated with decreased sperm concentration and vitality [11]. Of note, steroid hormone biogenesis from cholesterol (steroidogenesis) constitutes an important part of lipid homeostasis and plays a fundamental role in the maintenance and regulation of male fertility.

In the testis, balanced androgen biogenesis and androgen aromatization to estrogen is crucial [12,13]. It is generally accepted that interstitial Leydig cells are the main sites of aromatization in the adult gonad. Aromatase expressional changes can exist under abnormal conditions. For example, in patients with sex cord tumors (Peutz–Jeghers syndrome), Sertoli cells of seminiferous epithelium show non-physiologically high expression of this enzyme [14]. It is important to add here that estrogen action has been implicated in different physiological and pathological testis states [13,14,15,16,17]. From the above evidence, the question arises about the specific contribution of adipokines and aromatase in lipid status control in normal Leydig cells and tumor Leydig cells as well as to molecule—molecule interactions that can be a cause or repercussion of the tumor. Panza et al. [18] showed molecular modulation of aromatase activity that leads to inhibition of tumor growth in rats in vivo. In rat tumor Leydig cells (R2C) interplay between transcription factor dosage-sensitive sex reversal adrenal hypoplasia critical region on chromosome X gene 1 and androgens resulted in aromatase inhibition and decreased tumor cell proliferation [19]. Similarly, insulin growth factor 1 through steroidogenic factor 1 inhibited estrogen-dependent tumor Leydig cell proliferation [20].

Activation of lipid metabolism is an early event in tumorigenesis [21]; however, the precise expression pattern of lipid balance-controlling molecules and their molecular mechanism of action in pathological tissues is not fully characterized. In past decades, Leydig cell tumors were rare, constituting 1–3% of cases in adults and 4–9% of cases in prepubertal children. Nowadays, an increase in the incidences (14.7%) of this hormone-secreting tumor has been reported [22]. It is documented that Leydig cell tumors can secrete a variety of hormones e.g., testosterone, estrogen, and their derivatives [23]. Most Leydig cell tumors are benign but lately their malignant properties were reported [24,25]. In histology of this tumor different morphological types of steroidogenic cells as well as other cell types were observed [26]. In addition, Hibi et al. [27] reported the presence of adipocyte-like cells in tumor Leydig cell tissue.

Starting from the above findings, in this study, we investigated the expression of leptin, adiponectin and their receptors, and aromatase in human Leydig cell tumors. In addition, adipokine signaling proteins were examined to define the role these hormones play and whether there is a potential interaction between adipokines and aromatase. This study is a continuation of our prior reported data [28,29] and provides further knowledge on the biology of Leydig cell tumors.

## 2. Materials and Methods

### 2.1. Tissue Samples and Ethical Considerations

Residual tissues from testicular biopsy (microdissection testicular sperm extraction by Schlegel, [30]) were collected at the nOvum Fertility Clinic, Warsaw, Poland from patients (31–45 year–old; *n* = 20) diagnosed due to azoospermia (micronodules LCTs were recognized during surgery). After evaluation by pathologists and patient written informed consent according to the approval regulations by the National Commission of Bioethics at the Jagiellonian University in Krakow, Poland; permit no. 1072.6120.218.2017 and in accordance with the Declaration of Helsinki, specimens were used for the present study.

Tissue fragments were snap-frozen or fixed and paraffin-embedded, were stored and analyzed at the Department of Endocrinology, Institute of Zoology and Biomedical Research, Jagiellonian University in Krakow, Poland.

### 2.2. Body Fat Measurement

For body fat measurement, body mass index (BMI) based on height and weight of patients with the formula BMI = height (kg)/weight (m^2^) and reference categories according to National Institutes of Health, Bethesda, MD, USA website https://www.nhlbi.nih.gov was used.

### 2.3. Light andTtransmission Electron Microscopy Analyses

Tissues were immersed in ice-cold pre-fixative containing 2% formaldehyde and 2.5% glutaraldehyde in 0.1 M phosphate buffer, pH 7.3. The tissues were then rinsed and post-fixed in a mixture of 2% osmium tetroxide and 0.8% potassium ferrocyanide in the same buffer for 30 min at 4 °C. After dehydration in the graded series of ethanol and acetone, the material was infiltrated in a freshly prepared mixture of acetone and Epon 812 (Serva, Heidelberg, Germany) and embedded in Epon 812. Semi-thin sections (0.7 μm thick) were stained with 1% methylene blue and examined under a Leica DMR (Wetzlar, Germany) microscope. Ultrathin sections (80 nm thick) were contrasted with uranyl acetate and lead citrate and analyzed with a JEOL 2100 HT (Tokyo, Japan) TEM (for details see Bilinska et al. [31]).

### 2.4. Western Blotting

For quantification of protein expressions (Table 1) from LCTs proteins (as a control commercially available normal human Leydig cells; cat. No 10HU-103; ixCells Biotechnologies, San Diego CA, USA) were extracted in 50 μl of radioimmunoprecipitation assay buffer (RIPA; Thermo Scientific, Inc. Rockford IL, USA) and protease inhibitor cocktail (Sigma Chemical Co., St. Louis, MO, USA). Concentration of proteins was determined with Bradford reagent (Bio-Rad Protein Assay; Bio-Rad Laboratories GmbH, Munchen, Germany), using bovine serum albumin as a standard. Aliquots (50 μg protein) of cell lysates were used for electrophoresis on 12% mini gel by standard SDS-PAGE procedures under reducing conditions and electrotransferred to polyvinylidene difluoride (PVDF) membranes (Millipore Corporate, MA, USA) by a semi-dry transfer cell (Bio-Rad, Munchen, Germany). Then, blots were blocked with 5% nonfat dry milk in TBS, 0.1% Tween 20, overnight at 4 °C with shaking, followed by an incubation with respective antibodies (Table 1).

The membranes were washed and incubated with a secondary antibody conjugated with the horseradish-peroxidase labeled goat anti-mouse or goat anti-rabbit IgGs (Vector Labs., Burlingame, CA, USA) at a dilution 1:1000, for 1 h at RT. Immunoreactive proteins were detected by chemiluminescence with Western Blotting Luminol Reagent (Santa Cruz Biotechnology), and images were captured with a ChemiDoc XRS + System (Bio-Rad Laboratories). All immunoblots were stripped with stripping buffer containing 62.5 mM Tris–HCl, 100 mM 2-mercaptoethanol, and 2% SDS (pH 6.7) at 50 °C for 30 min and incubated with β-actin antibody. Each data point was normalized against its corresponding β-actin data point. Molecular masses were estimated by reference to standard proteins (Prestained SDS-PAGE Standards, Bio-Rad Labs, GmbH, Munchen, Germany). Quantitative analysis was performed for three separately repeated experiments using a public domain ImageJ software (National Institutes of Health, Bethesda, MD, USA) as described elsewhere [32]. The protein level within the control group was arbitrarily set as 1, against which the statistical significance of experimental groups was analyzed. The relative protein levels were expressed as arbitrary units.

### 2.5. Immunohistochemistry

To optimize immunohistochemical staining tissue sections both commercially available as a control (Zyagen, San Diego, CA, USA) and Leydig cell tumor sections were immersed in 10 mM citrate buffer (pH 6.0) and heated in a microwave oven (2 × 5 min, 700 W). Thereafter, sections were immersed sequentially in H_2_O_2_ (3%; *v/v*) for 10 min and normal goat serum (5%; *v/v*) for 30 min which were used as blocking solutions. After overnight incubation at 4 °C with primary antibodies listed in Table 1. Next respective biotinylated antibodies (anti-rabbit and anti-mouse IgGs; 1: 400; Vector, Burlingame CA, USA) and avidin-biotinylated horseradish peroxidase complex (ABC/HRP; 1:100; Vectastain Elite ABC Reagent, Vector Lab) were applied in succession. Bound antibody was visualized with 3,3′-diaminobenzidine (DAB) (0.05%; *v/v*; Sigma-Aldrich) as a chromogenic substrate. Controls included omission of primary antibody and substitution by irrelevant IgG.

### 2.6. Statistics

Three biological repeats of each sample and three independent experiments were performed. Each variable was tested using the Shapiro–Wilk W-test for normality. The homogeneity of variance was assessed with Levene’s test. Comparisons were performed by one-way ANOVA, followed by Dunnett’s post hoc test (GB-STAT software, v. 7.0; Dynamic Microsystems, Colesville, MD, USA) to determine the significant differences between proteins expression levels and BMI data. Statistical analyses were performed on raw data using Statistica 10 software (StatSoft Inc., Tulsa, OK, USA). Data were presented as means ± *S.D.* Data were considered statistically significant at * *p* < 0.05, ** *p* < 0.01 and *** *p* < 0.001.

## 3. Results

### 3.1. Body Mass Index in Patients with Leydig Cell Tumor

Body mass index measurement of patients revealed that only seven of them had body fat in the reference range (means = 23.85 ± 2.02 kg/m^2^) while thirteen patients were overweight (30.13 ± 1.78 kg/m^2^).

Due to no significant differences in Leydig cell tumor protein expression between non-obese and obese patients, the results are presented as combined. Only dissimilarities found in the tumor cell ultrastructure analysis are presented as separate for both groups of patients.

### 3.2. Topography and Ultrastructure of Leydig Cell Tumor

**Figure 1:**
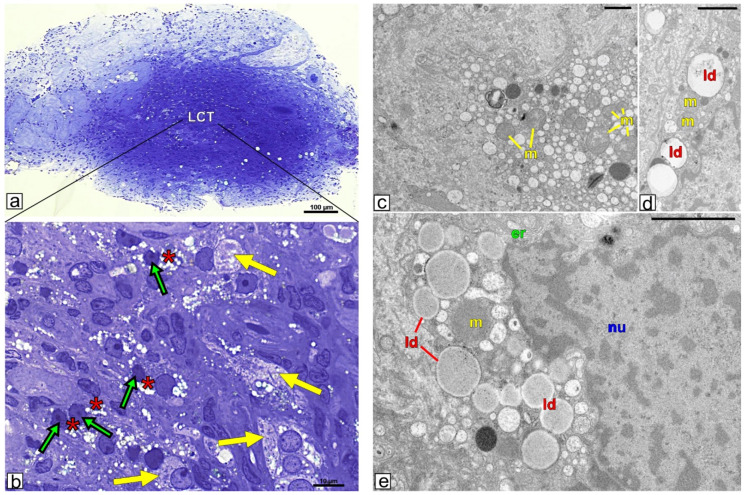
Ultrastructure of Leydig cell tumor. Representative microphotographs of semi-thin sections (**a** and **b** higher magnification) and ultrathin (**c**–**e**) of Leydig cell tumor. (**a**,**b**) Sections of testicular tissue of obese patient as representative for both obese and no-obese patients. Bars represent 1µm. (**c**–**e**) Sections of Leydig cell of obese patient (**c**) and non-obese (**d**,**e**) patient. Analysis was performed on testicular blocks from at least three experimental groups. (**a**,**b**) green arrows depict Leydig cells (different morphological types); red arrows depict adipocyte-resembling cells; asterisks indicate lipid droplets in Leydig cells. (**c**–**e)** ld-lipid droplets; m-mitochondria; *n*-nucleus; er-rough endoplasmic reticulum.

Leydig cell tumor mass was clearly resembled from testicular tissue without lesions (Figure 1a). The tumor cells were intensively stained by methylene blue. They were tightly packed and non-arranged, thus cell shape and size differed distinctly (Figure 1b). In cells of the tumor mass, abundant lipid droplet content was found (especially in obese patients; Figure 1a,b when compared to non-obese, not shown). In the majority of the cells, lipid droplets were present as groups of medium and/or large droplets in both obese and non-obese patients. In addition, small lipid droplets were occasionally seen. Besides cells with altered nucleus shape (many various shape alterations) large cells featuring adipocytes with spherical nucleus and lipid accumulation were also seen (Figure 1b).

Ultrastructural analysis confirmed apparently affected Leydig cells (Figure 1c–e). In addition to normal organelles a number of variously shaped and sized vesicles within the cytoplasm of tumor cells were observed especially in obese patients (Figure 1a) when compared to non-obese ones (Figure 1d,e). Careful analyses of the micrographs taken under the higher magnification suggest that at least some of those vesicles represent lipid droplets and/or in their content some lipids are present.

### 3.3. Expression of Leptin and Adiponectin and Their Receptors in Leydig Cell Tumor

**Figure 2:**
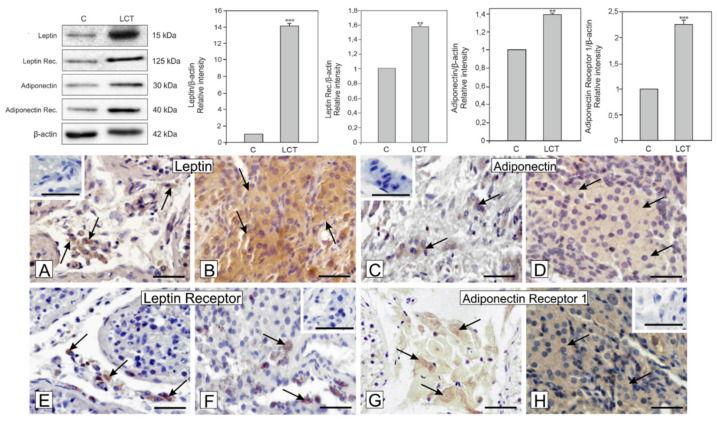
Expression (**upper panel**: blots and graphs) and localization of leptin and adiponectin and their receptors (**lower panel**: microphotographs) in a Leydig cell tumor. Representative blots and relative expression (arbitrary units) of qualitative expression of leptin, adiponectin, and their receptor proteins in Leydig cell of control (C) testis and Leydig cell tumor (LCT). The relative amount of respective proteins normalized to β-actin. Relative intensity of bands from three separate analyses is expressed as means. Asterisks show significant differences between expression of control and treated Leydig cells. Data is expressed as means ± S.D. Values are denoted as ** *p* < 0.01 and *** *p* < 0.001. Representative microphotographs of immunohistochemical localization of leptin (**A**,**B**), adiponectin (**C**,**D**), and their receptor proteins (**E**,**F** and **G**,**H**; respectively) in Leydig cell of control (C) testis and Leydig cell tumor (LCT). Counterstaining with hematoxylin. Bar 20µm. Inserts at (**A**,**C**,**F**,**H**)—negative controls. Immunostaining was performed at least three times. Arrows- depict positive staining.

Significantly increased expression of leptin (*p* < 0.001) and its receptor (*p* < 0.01) as well as adiponectin (*p* < 0.01) and its receptor (*p* < 0.001) was revealed in Leydig cell tumors when compared to control (Figure 2).

### 3.4. Localization of Leptin, Adiponectin and Their Receptors in Leydig Cell Tumor

A positive signal for leptin and adiponectin was found in the cytoplasm of Leydig cells of the control (Figure 2A,C). Signal of similar intensity as in respective control, either for leptin or for adiponectin was found in each of the cells of the tumor (Figure 2B,D). While positive signal for leptin receptor was present in every Leydig cell of control tissue, it was present occasionally in cells of the tumor (Figure 2E,F). On the contrary, the adiponectin receptor was present only in a few cells of control but in the Leydig cell tumor, all cells showed its expression (Figure 2G,H). No positive staining was visible when the primary antibodies were omitted (inserts at A,C,F,H).

### 3.5. Expression and Localization of Aromatase in Leydig Cell Tumor

**Figure 3:**
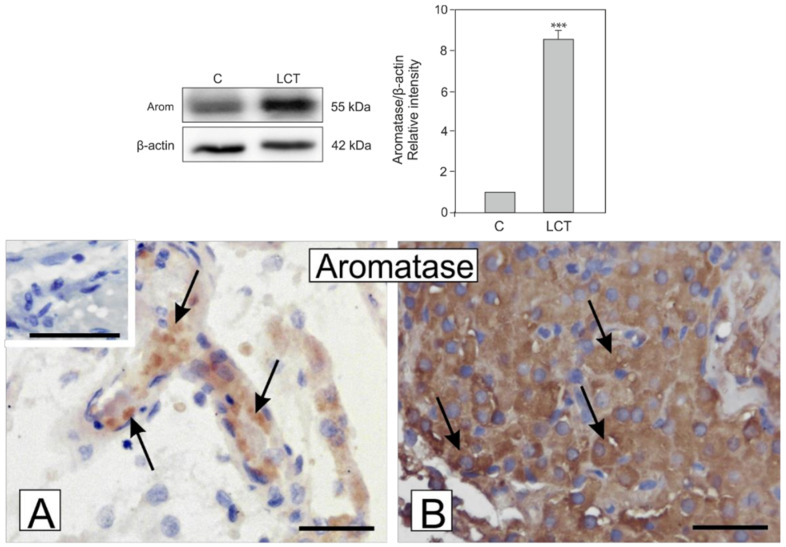
Expression (upper panel; blots and graph) and localization (lower panel; microphotographs) of aromatase in Leydig cell tumors. Representative blots and relative expression (arbitrary units) of qualitative expression of aromatase protein in Leydig cell of control testis (C) and a Leydig cell tumor (LCT). The relative amount of respective proteins normalized to β-actin. Relative intensity of bands from three separate analyses is expressed as means. Asterisks show significant differences between expression of control and treated Leydig cells. Data is expressed as means. Values are denoted as *** *p* < 0.001. Microphotographic documentation of DAB immunohistochemical localization of aromatase in Leydig cell of control testis (C) (**A**) and Leydig cell tumor (LCT) (**B**). Counterstaining with hematoxylin. Bar 20 µm. Insert at A—negative control. Immunostaining was performed at least three times. Arrows depict positive staining.

Marked increase in aromatase expression (*p* < 0.001) in Leydig cell tumors in comparison to control was found (Figure 3). In the Leydig cell tumor, very strong staining for aromatase when compared to the control where cells showed weak to moderate staining was observed (Figure 3a,b). No positive staining was visible when the primary antibody was omitted (insert at A).

### 3.6. Expression of Phospholipase C (PLC), Kinase Raf (Raf) and Extracellular signal-regulated kinase (ERK) in Leydig Cell Tumor

**Figure 4:**
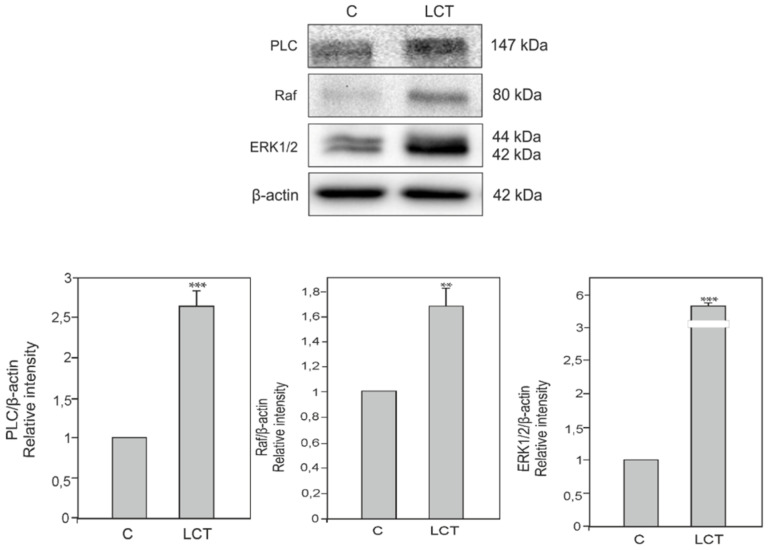
Expression of Phospholipase C (PLC), Kinase Raf (Raf) and Extracellular signal-regulated kinase (ERK) in Leydig cell tumors. Representative blots and relative expression (arbitrary units) of qualitative expression of PLC, Raf and ERK proteins in Leydig cell of control testis (C) and the Leydig cell tumor (LCT). The relative amount of respective proteins normalized to β-actin. Relative intensity of bands from three separate analyses is expressed as means. Asterisks show significant differences between expression of control and treated Leydig cells. Data is expressed as means. Values are denoted as ** *p* < 0.01 and *** *p* < 0.001.

Markedly increased expression of PLC (*p* < 0.001), Raf (*p* < 0.01), and ERK (*p* < 0.001) in the Leydig cell tumor when compared to control was revealed (Figure 4).

## 4. Discussion

Estrogens promote, maintain, and control the distribution of adipose tissue and its metabolism. These hormones are potent anorectic agents reducing food intake and body weight. The feeding behavior was reported to be based on estrogen receptor α (ERα) signaling in hypothalamic nuclei [33]. In ovariectomized rodents, estrogen similarly to leptin affected food intake and body weight [34]. Sex-dependent differences in levels of circulating leptin are well described [35]. In women, the concentration of leptin is always higher than in men [36]. Moreover, Tanaka et al. [37] demonstrated that estrogen application elevated the circulating leptin concentration in both humans and rats and increased leptin mRNA expression in adipose tissue in either in vitro or in vivo conditions. Adiponectin is one of adipokines that protects against obesity-related diseases in premenopausal women [38]. It was showed that oophorectomy of adult mice increased adiponectin level and reversed it by estradiol replacement [39]. On the other hand, the effect of adiponectin on bone metabolism is effectively suppressed by estradiol treatment [40].

Many lines of evidence confirmed the expression of both leptin and adiponectin in the interstitial tissue of rodent and human testis [41,42,43,44]. High expression of leptin receptor was correlated with the serum testosterone level [41]. New data by Panza et al. [18] indicated leptin as a key factor able to affect testicular seminoma progression, and its receptor as a potential target for novel treatments in this type of cancer. The study found high expression of leptin, adiponectin and their receptors in Leydig cell tumors, which can be considered as possible new diagnostics and therapeutic markers. However, no pronounced differences in expression of proteins studied here were found between those used for the study: tumor testicular tissues of azoospermic non-obese, and obese patients. It should be added that previously we reported increased perilipin expression and decreased steroidogenic acute regulating protein expression in these patients [27]. In addition, increased estradiol levels can reflect increased cholesterol content and/or perturbations in its processing in men with Leydig cell tumors. Epidemiological studies have highlighted associations between increased serum leptin levels and increased tumor growth, while adiponectin exhibited an inverse correlation with the development and progression of various cancers [45]. In addition, high expression of adipokines in Leydig cell tumors indicates intensive tumor metabolism that may lead to fast tumor growth and molecular changes, and cannot be excluded here. It was demonstrated that in the testis, adipokines regulate metabolism through both paracrine and autocrine action [46,47]. Low expression of peroxisome-proliferator activated receptor α, β, and γ (PPAR α, β, and γ) was confirmed in the Leydig cell tumor [28] and its regulation by adiponectin was confirmed [48]. Our recent findings showed different morphological types of Leydig cells present in tumor mass [28]. Herein, on ultrathin sections of the tumor, we confirmed also the presence of adipocyte-like cells. Of note, adipocyte-like cells can be an additional source of adipokines that signal in the tumor microenvironment. Tumor Leydig cells exhibited accumulation of lipids, which show subtle differences in their size and distribution in non-obese and obese patients visible at the ultrastructural level. Therefore, azoospermia in patients studied here may be a result of both Leydig cell dysfunction with involvement of increased body fat mass and lipid homeostasis-regulating hormones. It seems to be crucial to design a study that in detail will examine the mechanism and regulation of these pathological processes. Lipids and their metabolisms can have an effect on spermatogenesis and/or its efficiency, as well as both Leydig cell tumor development and its diagnosis/therapy. Obesity is one of the factors that impacts malignant breast tumor progression leading to alterations in levels of hormones, growth factors, and inflammatory markers e.g., cytokines and adipokines [49]. Moreover, recent findings by Hoffmann et al. [50] and Shuang et al. [51] in ovarian cancer cells report proliferation regulation by adiponectin through reversing effect of estradiol and enhancing proliferation by other adipokines. Stimulatory effect of leptin on cell growth and proliferation was observed in normal and cancer cells of the prostate [52]. On the basis of above facts, we postulate that adipokines alone or with estradiol can form pathological hormonal microenvironment effecting on mitosis resuming by Leydig cells in adult testis. In fact, healthy mature Leydig cells of adult lineage do not proliferate [53]. However, last finding revealed in vitro proliferation of sheep adult Leydig cells after metalloestrogen (selenium) treatment [54].

Concomitantly, in this study increased expression of leptin, adiponectin and their receptors as well as increased expression of aromatase was revealed. In our previous study, we reported an increased concentration of estradiol in Leydig cell tumors [55]. According to findings by Carpino et al. [14] the site of aromatization is restricted to and increased in the cell types that are compromised in the disease, as evidenced in Sertoli and Leydig cell tumors, and germ cell neoplasia. The localization of aromatase in altered cells suggests there are additional sources of estrogen production in the testis, which may function to cover tumor feed, energetic and metabolic needs, thus affecting its growth and development of further malignant and metastatic properties through maintaining of the local tumor microenvironment [49].

It was shown that expression of aromatase is proportional to body fat mass and causes more fat accumulation, thus forming a vicious cycle [56]. Excessive aromatase activity in adipose tissue leads to disturbed androgen/estrogen balance underlying obesity-related infertility. However, we were not able to reveal subtle differences in aromatase expression that might exist in tumor tissue between non-obese and obese patients. The effect of estrogens in testicular somatic cells is inhibitory, leading to reduced testosterone levels and sperm production; however, it has been observed that aromatase participates in the acquisition of sperm motility [56]. Obesity and the resulting chronic inflammatory state (we cannot ignore the possibility of the presence of cells of the immunological system in Leydig cell tumor masses) increase aromatase expression and estrogen synthesis. In the aromatase knockout (ArKO) mouse, levels of leptin are increasing within the time, along with two to four times more visceral adiposity [57]. The increase in aromatase immunoreactivity is observed during testis gestational development and it coincides with the period of Leydig cell precursors hyperplasia, a normal part of testis development essential to cover steroidogenic needs of the human fetal testis [58]. It seems also possible that in Leydig cell tumors, an increase of aromatase expression is responsible for lipid metabolism disturbance and in turn tumor transformation of normal cells. In fact, treatment with aromatase inhibitors is one of the therapeutic trials in women with breast carcinoma that affects lipid metabolism and inflammatory response [59].

What is more, in human Leydig cell tumors, varying expression patterns of both ER and non-canonical G-protein coupled estrogen receptor were observed [14,28,29,60]. These findings suggest that altered estrogen signalization with canonical and non-canonical estrogen receptors is involved in the biochemical properties of Leydig cell tumors and it should be under consideration as an important treatment target. In addition, attribution of the changes in organelle structure and function to estrogen and/or adipokine and/or lipid level and signaling also should be given special attention.

The mechanisms underlying the regulation of leptin and adiponectin concentrations by estrogen are not well-recognized. We showed for the first time that aromatase as well as phospholipase C (PLC) and kinases: Raf, ERK (extracellular signal–regulated kinase) can be part of adipokine signaling pathways in human Leydig cell tumors. It was found that estrogen enhanced leptin promoter activity in human placenta choriocarcinoma cell lines (JEG-3) [61]. Gambino et al. [62] demonstrated that estrogen-induced leptin expression in trophoblast cells through genomic and non-genomic mechanisms involved ERα. In adipocytes cell line (3T3-L1), ERα induced leptin expression whereas ERβ inhibited it [63]. The ratio of ERα to ERβ expression may be an important potential regulatory factor in leptin expression, but it is disturbed in Leydig cell tumors [for review see 28,29]. We found an increased expression of PLC, Raf kinase, and ERK, that can indicate changes in signaling pathways involved and/or interacting with pleiotropic adipokine signaling [64]. In leptin-deficient mice PLC acted via phosphoinositide 3-kinase [65] while in muscle cells, adiponectin acted directly through PLC pathway [66] and in vascular smooth muscle cells, adiponectin used Raf-ERK pathway to regulate proliferation and apoptosis [45]. Data of the present study reflect that both proliferation and apoptosis occur in cells of the tumor and they are controlled via leptin and adiponectin together with PLC, Raf, and ERK. It was demonstrated that cancers acquire genetic mutations and epigenetic modifications that can result in the activation of oncogenes e.g., Raf and ERK, or can lead to inactivation of tumor suppressors [67]. Of note, through ERK pathway leptin influenced cumulus maturation and steroidogenic secretion in rabbit [68] and adiponectin in goat [69]. As mentioned above, incomplete data reflects distinct adipokine signaling pathways and/or single molecules of adipokine signaling can take part in Leydig cell regulation under central hypothalamus-pituitary-gonadal axis and via local lipid- and estrogen- created microenvironment [70,71].

## 5. Conclusions

Based on these results, we suggest that there is a link between adipokines and aromatase (estrogens) levels, signaling and adipokine-, estrogen- signaling interactions in Leydig cell tumors. Increased aromatase expression seems to be both cause and consequence of Leydig cell tumor initiation and/or progression. In Leydig tumor cells, aromatase and secreted estrogens can affect adipokine levels in the testicular interstitial tissue and/or body fat tissue leading to fertility disturbances. Elevated adipokine signaling with the implication of various transductional pathways in Leydig cell tumors showed that adipokines are also pleiotropic hormones in the pathological testis. Their interactions via endocrine/paracrine/autocrine pathways may contribute to the hormone (including estrogen) dependence of testicular tumorigenesis by stimulating Leydig cell proliferation, growth, and malignancy. Leptin and adiponectin, as well as estrogen production and their transductional routes, seem to be important for further considerations as potential targets for diagnosis and therapy of Leydig cell tumors and adipose tissue dysfunction [11,72], although more precise analyses e.g., ultrastructural and epigenetical, should be used for precise recognition of subtle differences that can exist in tumor tissue biochemistry between non-obese and obese patients. What is more, special attention should be paid to an explanation of the association of adiponectin, leptin, with infertility and BMI as it is still controversial [73,74].

## Figures and Tables

**Table 1:** Primary antibodies used for Western blot and immunohistochemistry.

Antibody	Host Species	Vendor	Dilution
adiponectin	goat	Santa Cruz Biotechnology cat no. sc-26496	1:50 (IHC) 1:500 (WB)
adiponectin receptor 1	goat	Santa Cruz Biotechnology cat no. sc-46749	1:50 (IHC) 1:500 (WB)
cytochrome P450 aromatase	mouse	Bio-Rad cat no. MCA2077S	1:50 (IHC) 1:500 (WB)
leptin	mouse	Sigma–Aldrich cat. no. L3160	1:50 (IHC) 1:500 (WB)
leptin receptor	mouse	Santa Cruz Biotechnology cat. no. sc-8391	1:50 (IHC) 1:500 (WB)
phospholipase C (PLC)	mouse	Abcam cat. no. ab243181	1:500 (WB)
kinase Raf	rabbit	Cell Signaling Technology cat. no. 9422	1:500 (WB)
extracellular signal-regulated kinase (ERK1/2)	rabbit	Cell Signaling Technology cat. no. 9102	1:1000 (WB)
β-actin	mouse	Sigma–Aldrich cat. no. A2228	1:3000 (WB)
